# Environmental effects on protection against *Mycobacterium tuberculosis* after immunization with Ad85A

**DOI:** 10.1016/j.vaccine.2012.12.024

**Published:** 2013-02-04

**Authors:** Peter Beverley, Edward Ronan, Lianni Lee, Isabelle Arnold, Beatrice Bolinger, Fiona Powrie, Elma Tchilian

**Affiliations:** a University of Oxford, The Peter Medawar Building for Pathogen Research, South Parks Road, Oxford OX1 3SY, UK; b University of Oxford, Translational Gastroenterology Unit, John Radcliffe Hospital, Oxford OX3 9DU, UK

**Keywords:** Tuberculosis, Antigen 85A, Adenovirus, Subunit vaccine, Environmental mycobacteria

## Abstract

Previously we have shown that intradermal (i.d.) immunization with a recombinant adenovirus expressing antigen 85A (Ad85A) induced a strong splenic CD8 T cell response in BALB/c mice but a weak lung immune response and did not protect mice against challenge with *Mycobacterium tuberculosis (Mtb)*. After moving to a new animal house, the same i.d. immunization induced a strong lung immune response and the mice were protected against *Mtb* challenge. Increased numbers of antigen 85A-specific CD8 cells were present in lung tissue but were not recoverable by bronchoalveolar lavage (BAL). Mycobacterial growth was inhibited 21 days after *Mtb* challenge. In contrast, the effects of intranasal (i.n.) immunization did not change between the animal houses; 85A-specific T cells were recovered by BAL and were able to inhibit *Mtb* growth early after challenge. The effect of alterations to the environment was investigated by administering BCG or *Mycobacterium abscessus* in the drinking water, which induced protection against *Mtb* challenge, while *Mycobacterium smegmatis* did not. However, when Ad85A was given i.d. at the same time as BCG or *M. abscessus*, but not *M. smegmatis*, the protection induced by Ad85A was abolished. Treatment of mice with a CD25 antibody during the challenge period, abolished the suppressive effect of oral mycobacterial administration, suggesting that regulatory T cells (T regs) were involved. These results showed that exposure to environmental microorganisms can alter the protective immune response to a parenterally administered subunit vaccine, a result with important implications for the use of such vaccines in humans.

## Highlights

► The environment alters the immune response to an intradermal (i.d.) TB subunit vaccine, Ad85A. ► Ad85A i.d. induces a lung immune response but is less protective against *Mtb* than intranasal Ad85A. ► Oral administration of environmental mycobacteria reduces Ad85A i.d induced protection. ► *In vivo* depletion of CD25+ regulatory T cells abolishes the effect of oral mycobacteria.

## 1 Introduction

Tuberculosis (TB) remains an important healthcare problem causing 1.5 million deaths a year. An effective vaccine would alleviate the problem but in spite of much effort, BCG remains the only licensed vaccine. BCG protective efficacy varies widely in different geographical areas [1], perhaps because of varying exposure to environmental mycobacteria (EM). Immunity to EM may inhibit BCG multiplication and prevent the induction of an efficient protective immune response [2]. Alternatively EM-induced immunity may mask BCG induced protection [3] or induce regulatory T cells (T regs) [4].

We have used Ad85A, to explore the contributions of local and systemic immunity to protection against *Mtb*. In BALB/c mice this vaccine induces a powerful CD8 T cell response and weak CD4 response and there is strong published evidence that the CD8 T cells mediate protection [5,6].

Using this model we have shown that both local pulmonary and systemic immunity are important in protective immunity against *Mtb.* Local immunity inhibits the growth of *Mtb* very early after challenge infection, while systemic immunity acts later. Furthermore, local and systemic immunity can have additive protective effects [7,8]. Induction of local immunity might therefore be a useful strategy for future *Mtb* vaccines. However, lung as well as systemic immunity is affected by environmental microbial exposure. Thus for example, infection with Helicobacter can suppress allergic lung responses [9] and responses to influenza virus can also be modulated [10], although the effects of microbial exposure on lung immune responses to parenterally administered *Mtb* subunit vaccines have not been investigated.

Our earlier experiments on immune response and protection induced by i.d. and i.n. administration of Ad85A were carried out using mice supplied as specific pathogen free (SPF) animals but maintained in the animal house in open top cages (OTC) [7,11]. When we moved to a new facility, in which identical mice were housed in individually ventilated cages (IVCs), we found that both immune responses and protection induced by the same vaccine were profoundly altered. Whereas previously Ad85A i.d. generated a strong splenic, but only a very weak lung immune response and the mice were not protected against challenge [11], after moving, we observed that Ad85A i.d. induced a strong antigen-specific CD8 T cell response in the lungs. The immune response and protection induced by Ad85A i.n. were unaffected by the change of animal house. As most of the subunit *Mtb* vaccines that are in advanced clinical trials are administered parenterally [12] we investigated whether the altered immune response to Ad85A i.d. correlated with protection and why lung immune responses of the same strain of mice, immunized with the same vaccine, differed in the two animal houses.

## 2 Materials and methods

### 2.1 Mice and immunization

All experiments were performed with 6–8 week old female SPF BALB/c mice (Harlan Orlac, Blackthorn, UK), were approved by the animal use ethical committee of Oxford University and complied with UK Home Office guidelines. In one animal facility, mice were housed in OTCs and drank tap water. In the second facility, the mice were kept in IVCs and given water purified by reverse osmosis.

For i.d. immunization, BALB/c mice were anesthetized with isofluorane and injected with 25 μl PBS in each ear, containing 2 × 10^9^ virus particles of Ad85A per mouse, and for i.n. immunization allowed slowly to inhale 40 μl of PBS containing 2 × 10^9^ virus particles of Ad85A [7].

T regs were depleted by 3 intraperitoneal (i.p) injections of 80 μg anti-CD25 antibody (clone PC61) one day before and 7 and 14 days after *Mtb* challenge. Control mice received 80 μg normal rat Ig (Abcam, Cambridge, UK).

### 2.2 Oral exposure to mycobacteria

BCG (Statens Serum Institute, Copenhagen, Denmark), *Mycobacterium abscessus* (kindly provided by Dr Sandra Newton, Imperial College, London) or *Mycobacterium smegmatis* (kindly provided by Dr Kathryn Lougheed, Imperial College, London) were administered in the drinking water. *M. abscessus* and *M. smegmatis* were cultured in 25 ml LB broth supplemented with 10% OADC (oleic acid, albumin, dextrose and catalase, Sigma) at 37 °C with shaking. After 48 h the cultures were centrifuged at 600 × *g* for 10 min and the pellets resuspended in 500 ml of reverse osmosis water in the cage bottles. Ten vials of BCG (SSI) were similarly suspended in 500 ml of water. Mycobacterial colony forming units (CFU) were enumerated by plating on 7H11 Middlebrook agar plates (E&O Laboratories Ltd., Bonnybridge, UK) and counting colonies after incubation. Mycobacterial concentration was between 10^3^ and 10^4^ CFU/ml of water. After 1 week, *M. abscessus* and *M. smegmatis* remained at the same concentration, however no viable BCG was recovered. Water bottles containing mycobacteria were changed weekly.

### 2.3 Isolation of lymphocytes from lungs, BAL and spleen

Lungs were perfused with PBS, cut into pieces and digested with 0.7 mg/ml collagenase type I (Sigma) and 30 μg/ml DNase I (Sigma) for 45 min at 37 °C. Digested fragments were crushed through a cell strainer using a syringe plunger, washed, layered over Lympholyte (Cederlane, Ontario, Canada) and centrifuged at 1000 × *g* for 25 min. Interface cells were collected and washed. BAL was collected by washing *via* the trachea. The collected cells were centrifuged and resuspended in medium. Spleens were passed through a cell strainer using a syringe plunger, red blood cells were removed with lysis buffer (Qiagen, Crawley, UK) and the cells were washed.

### 2.4 Flow cytometry

Cells were cultured in Hepes buffered RPMI, 10% heat-inactivated FCS, l-glutamine, penicillin and streptomycin for 6 h. Cells from Ad85A immunized animals were stimulated for 6 h with either 66 15-mer peptides overlapping by 10 amino acids covering the 85A protein sequence or with 3 peptides (Peptide Protein Research Ltd., Fareham, UK) encoding the dominant CD4 (Ag85A_99–118aa_ TFLTSELPGWLQANRHVKPT) and CD8 (Ag85A_70–78aa_ MPVGGQSSF and Ag85A_145–152aa_ YAGAMSGL) peptide epitopes [7] (Peptide Protein Research Ltd.). Each peptide was 2 at μg/ml. After 2 h at 37 °C, Golgi Plug (BD Biosciences, Oxford, UK) was added according to the manufacturer's instructions.

Cells were washed and incubated with CD16/CD32 monoclonal antibody to block Fc binding. Subsequently the cells were stained for CD4 (RM4-5), CD8 (53-6.7) (BD Bioscience, Oxford, UK), anti I-A/I-E (2G9), CD25 (PC61.5), CD11c (HL3), then for intracellular FoxP3 (FJK-16s), IFNγ (XMG1.2), IL-2 (JES6-5H4) and TNF (MP6-XT22) (eBioscience, Hatfield, UK) using the BD Cytofix/Cytoperm kit according to the manufacturer's instructions. Cells were fixed with PBS + 1% paraformaldehyde, run on a LSRII (BD Biosciences) and analyzed using FlowJo software (Tree Star Inc., Ashland, Oregon, USA).

### 2.5 Infection with *Mtb* and determination of mycobacterial load

Five to 7 mice were anesthetized with isoflurane and infected i.n. with *Mtb* (Erdman strain, kindly provided by Dr Amy Yang, CBER/FDA) in 40 μl PBS. Lung CFU were enumerated 24 h after challenge to determine the number of organisms deposited (∼200 CFU). Mice were sacrificed at indicated times, the lungs and spleen homogenized and mycobacterial load determined by plating 10-fold serial dilutions of tissue homogenates on Middlebrook 7H11 agar plates (E&O Laboratories Ltd., Bonnybridge, UK). Colonies were counted after 3–4 weeks of incubation at 37 °C in 5% CO_2_.

### 2.6 Statistical analysis

Data were analyzed using one-way ANOVA followed by Tukey's multiple comparison test. Immune responses were assessed using Mann Whitney test.

## 3 Results

### 3.1 Effect of the environment on Ad85A i.d. induced immune responses and protection

**Fig. 1:**
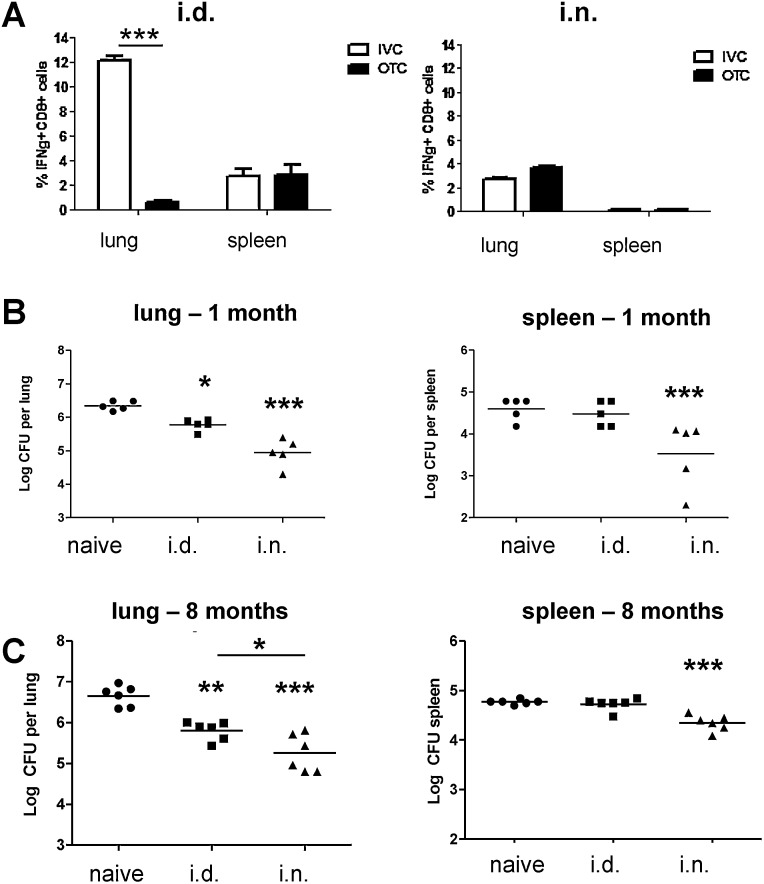
T cells responses and protection against *Mtb* in different animal facilities. (A) Cytokine responses of T cells to antigen 85A. BALB/c mice were immunized with Ad85A i.d. or i.n. in an animal house with open top cages (OTC) or an animal house with individually ventilated cages (IVC). Lung and splenic lymphocytes were isolated 5 weeks post immunization and stimulated with pooled 85A peptides. The percentage of CD8+ cells expressing IFNγ was determined by flow cytometry. Error bars show SD. (B) *Mtb* growth after Ad85A i.d. or i.n. immunization in IVCs. In two experiments, BALB/c mice were immunized with Ad85A i.d. or Ad85A i.n. One month (B) or 8 months (C) later they were challenged with *Mtb* and lung CFU enumerated 5 weeks later. Data from the two experiments with 5–7 mice/group are shown. ***p < 0.001, **p < 0.01, *p < 0.05 from naïve or between the indicated groups, one-way ANOVA with Tukey's post test.

The responses of mice immunized with Ad85A i.d. or i.n. in OTCs or IVCs were compared, although the experiments could not be performed simultaneously because the OTC facility had been demolished by the time experiments were performed in the IVC facility. IVC mice immunized with Ad85A i.d. made a very strong lung 85A-specific CD8 T cell response, with ∼12% of CD8 cells producing IFNγ, compared to 0.7% for OTC Ad85A i.d. immunized mice (Fig. 1A) [6,11,13]. There was no increase in the number of lung lymphocytes and percentage of CD8 T cells in IVC Ad85A i.d. compared to unimmunized mice.

The lung response to Ad85A i.n. and splenic responses to the vaccine given by either route were unchanged (Fig. 1A), as were CD4 responses, which were always low for this vaccine and strain of mice [5,7,11]. Although Ad85A i.d. induced a much stronger lung CD8 response in IVCs than Ad85A i.n. (12.1% i.d. *versus* 2.8% i.n.), the proportions of single, double or triple IL-2, TNF and IFNγ cytokine producing cells, did not differ greatly between mice immunized by the two routes, as shown previously [7,11].

When mice were immunized in OTC, the magnitude of lung but not splenic immune responses, correlated with protection after Ad85A immunization [11]. We therefore tested whether this was the case in IVCs. Ad85A i.d. or i.n. mice were challenged 5 weeks or 8 months after immunization and *Mtb* lung CFU assessed 5 weeks later. The lung mycobacterial load was reduced by 0.8 log_10_ in Ad85A i.d. immunized compared to naïve animals, while Ad85A i.n. mice showed a 1.5 log_10_ reduction (Fig. 1B). Protection of the lungs after Ad85A i.d. or Ad85A i.n. was sustained for up to 8 months (Fig. 1C). The effect of Ad85A i.d. on spleen CFU was inconsistent, with no reduction in the experiments shown in Fig. 1B and C, but a reduction in others (Fig. 3A). In contrast Ad85A i.n. consistently reduced splenic CFU, suggesting that i.n. immunization may reduce dissemination as well as lung *Mtb* growth.

These data showed that environmental changes profoundly altered the lung antigen-specific response and protection after Ad85A i.d. immunization, and confirmed that lung immune responses, although not their magnitude, correlated with protection after Ad85A immunization. For the first time we showed that Ad85A i.d. could induce sustained protection, although protection afforded by Ad85A i.n. was always superior, despite the higher lung immune response to Ad85A i.d.

### 3.2 The location of antigen-specific cells and kinetics of *Mtb* growth after Ad85A i.d. and Ad85A i.n. immunization

Previously, intranasal immunization with Ad85A or recombinant 85A protein, was shown to inhibit *Mtb* growth in the first week after challenge and antigen specific-cells were found in BAL, while effective parenteral vaccines inhibited *Mtb* growth later and no antigen specific cells were found in BAL [6–8]. We therefore analyzed BAL in Ad85A i.d. and i.n. animals maintained in IVCs and determined the kinetics of *Mtb* growth after challenge.

**Fig. 2:**
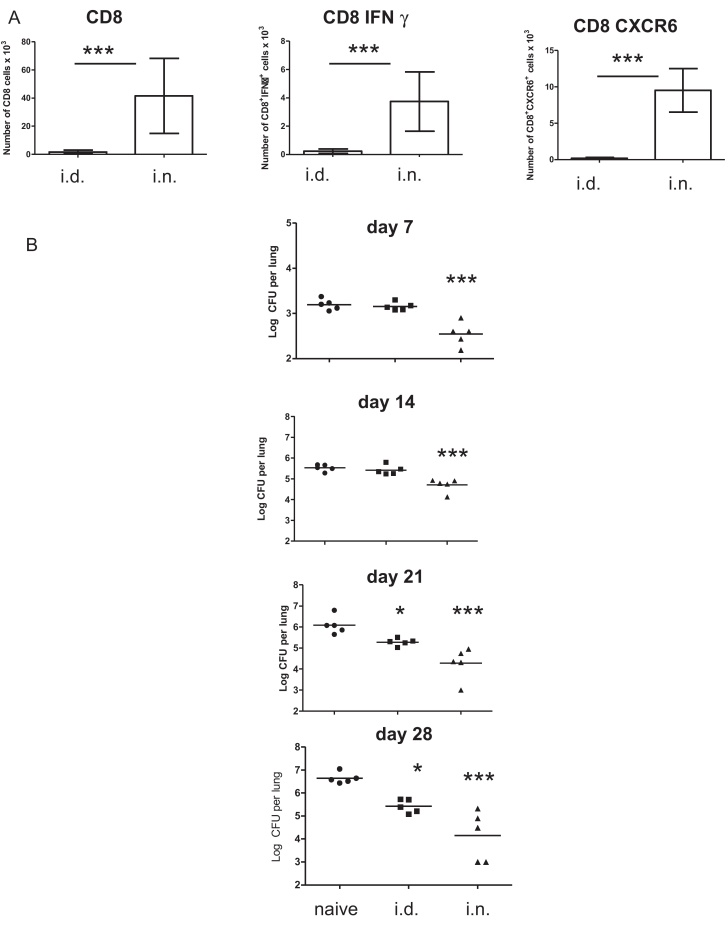
Location of cells and kinetics of *Mtb* growth after Ad85A immunization. (A) BALB/c mice in IVCs were immunized with Ad85A i.d. or i.n. Four weeks post immunization BAL was collected from individual mice and the number of CD8+, CD8IFNγ+ and CD8CXCR6+ cells determined by flow cytometry. The figures show the mean number of each cell population recovered ±SD from 3 mice in one of three independent experiments ***p ≤ 0.005. (B) Kinetics of *Mtb* growth after Ad85A i.d. and i.n. immunization. BALB/C mice were immunized in IVCs once with Ad85A i.d. or i.n. Five weeks after immunization mice were challenged with *Mtb* and groups of 5 mice sacrificed 7, 14, 21 and 28 days later for enumeration of lung *Mtb* CFU. ***p < 0.001, *p < 0.05 compared to naïve animals, one-way ANOVA with Tukey's post test. Representative data from one of two experiments are shown.

BAL was collected from Ad85A i.n. and Ad85A i.d. mice 4 weeks post-immunization. CD8+ T cells were abundant in BAL of Ad85A i.n. but scanty in BAL of Ad85A i.d. mice. In Ad85A i.n. mice, ∼10% of BAL CD8 cells were 85A-specific producing IFNγ in response to stimulation with 85A peptides, and many expressed CXCR6+ (Fig. 2A) [6–8]. Very few antigen specific T cells were found in BAL of Ad85A i.d. mice, even though a greater proportion of 85A specific CD8 T cells were found in lung tissue (Fig. 1A). Thus the localization of 85A-specific cells differed between Ad85A i.n. and i.d. immunized mice.

To examine the kinetics of mycobacterial growth, Ad85A i.n. or Ad85A i.d. mice were sacrificed 7, 14, 21 and 28 days post *Mtb* challenge. As before [7], Ad85A i.n. mice inhibited *Mtb* growth at day 7, while i.d. animals did not do so until day 21 (Fig. 2B). Thus although both Ad85A i.d. and i.n. animals had antigen specific cells in the lungs (Fig. 1A), only Ad85A i.n. mice had 85A-specific cells in BAL and inhibited early *Mtb* growth (Fig. 2A and B).

### 3.3 Effect of mycobacterial exposure on Ad85A i.d. induced protection

Microbial exposure of mice in OTCs drinking tap water, is likely to differ from mice in IVCs drinking purified water. We analyzed immune responses to Ad85A i.d. in several OTC and IVC facilities, but in all observed large lung responses, suggesting that the original facility had some unique environmental property. However, this is not the first time that unique responses of mice from one animal house have been described, as C57BL/6 J mice differ in intestinal flora and in the number of Th17 cells in the intestine, from C57BL/6 mice from all other suppliers [14]. Therefore, reasoning that microbial exposure, most likely in drinking water might explain the effects of our former facility, we tested the effect of different mycobacteria administered orally on protection induced by Ad85A i.d.

**Fig. 3:**
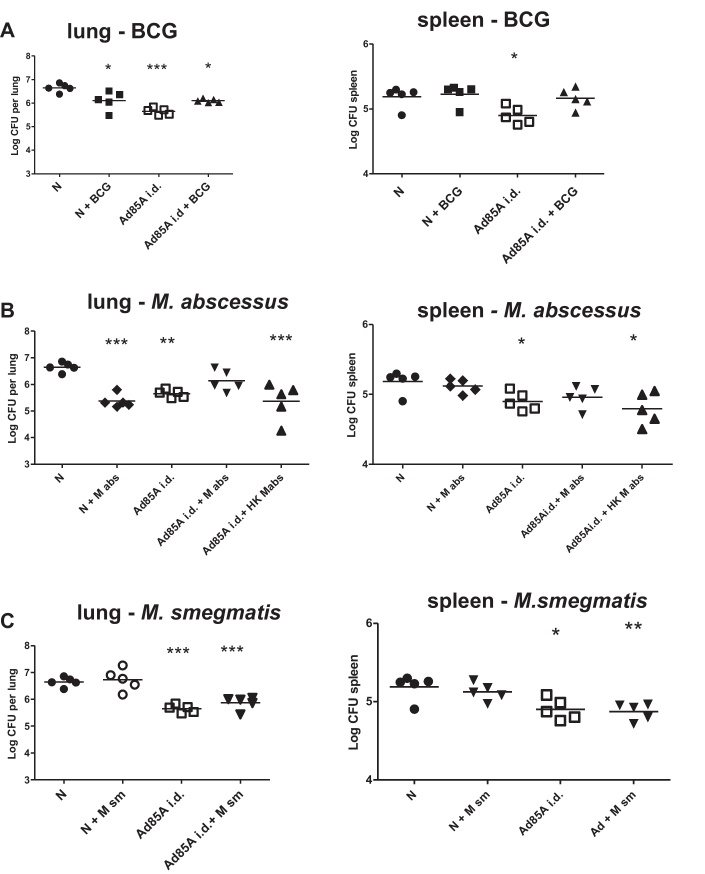
Effect of oral mycobacteria on Ad85A i.d. induced protection. Unimmunized (N) or Ad85A i.d. immunized BALB/c mice in IVCs were given drinking water with or without BCG (A), *M. abscessus* (M abs) or heat killed *M. abscessus* (HK M abs) (B) or *M. smegmatis* (M sm) (C). Five weeks after Ad85A i.d. immunization they were challenged with *Mtb* i.n. and after further 5 weeks sacrificed for enumeration of lung and spleen *Mtb* CFU. Data from one of two experiments with 5–7 mice/group are shown. ***p < 0.001, **p < 0.01, *p < 0.05 compared to naïve animals with clean water, one-way ANOVA with Tukey's post test.

Mice were immunized with Ad85A i.d. and given BCG in the drinking water from the day of immunization until challenged with *Mtb* after 5 weeks. Unimmunized mice were also given BCG water. Unimmunized animals given BCG water showed 0.6 log_10_ CFU less than controls with clean water (Fig. 3A), in accordance with evidence that oral BCG induces protective immunity [15]. Ad85A i.d. alone also reduced *Mtb* growth by 0.9 log_10_. Strikingly Ad85A i.d. and BCG water were not additive, but instead *Mtb* growth was the same as in mice given BCG water alone (0.6 log_10_). BCG water alone did not affect spleen CFU, although together with Ad85A i.d. it reversed the splenic protective effect of Ad85A i.d.

We next tested *M. abscessus*, which is found in water and this alone reduced lung CFU by 1.3 log_10_ (Fig. 3B) but like BCG, abolished the protection afforded by Ad85A i.d. Heat killed *M. abscessus* or live *M. smegmatis* in the water did not protect against *Mtb* nor did they affect protection induced by Ad85A i.d. (Fig. 3B and C).

To investigate the mechanisms of these effects on Ad85A i.d. induced protection, we measured 85A-specific responses in the lungs and spleen 4–5 weeks after immunization. In 2 out of 4 experiments there were reduced numbers of 85A-specific CD8 T cells in the lungs of Ad85A i.d. mice given BCG or *M. abscessus* water but no change in spleen responses (not shown). The supernatants of 48 h lung and spleen cell cultures stimulated with 85A peptides were also assayed for cytokines, but there were no consistent differences between mice given only Ad85A i.d. and those given Ad85A with mycobacteria in the water (not shown).

### 3.4 Depletion of T regs abolishes the inhibitory effect of *M. abscessus* on Ad85A i.d. protection

Exposure to antigens in the gut induces regulatory T cells (T regs) and T regs have been shown to inhibit protective immune responses to *Mtb* [16–18]. We therefore used CD25 antibody, given the day before and 7 and 14 days after *Mtb* challenge, to deplete T regs from immunized and mycobacteria-exposed mice. Two days after the first administration of CD25 antibody, CD4+CD25+Foxp3+ cells decreased by ∼55% in the lungs and spleen and 4 days after the second administration by ∼68%, indicating a sustained depletion of CD4+CD25+Foxp3+ T regs, a result similar to those obtained by others [19]. Control rat Ig had no effect.

**Fig. 4:**
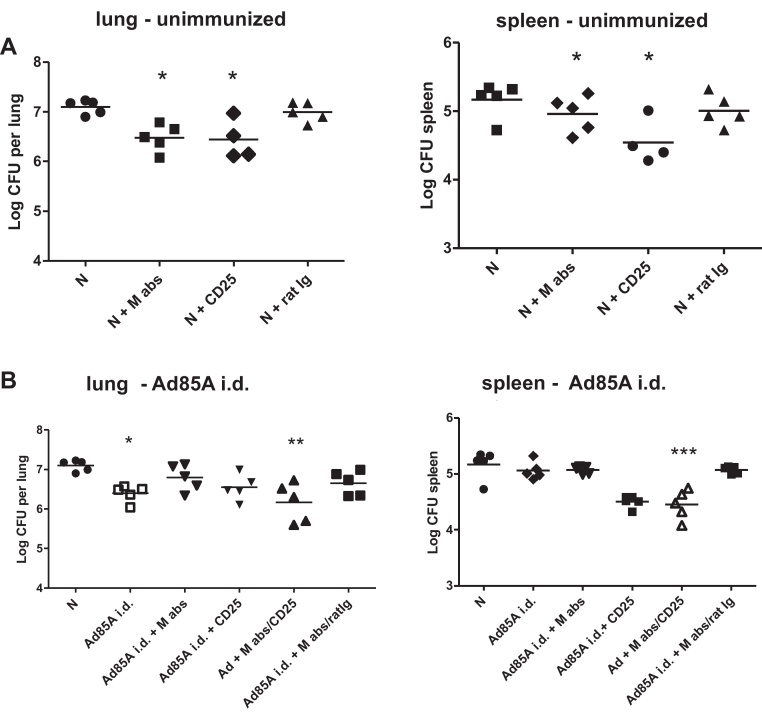
*Mtb* CFU after CD25 depletion. Unimmunized (N) or Ad85A i.d. immunized BALB/c mice in IVCs were given drinking water with or without *M. abscessus* (M abs). Five weeks after the immunization they were challenged with *Mtb* i.n. and after further 5 weeks sacrificed for enumeration of lung and spleen *Mtb* CFU. The day before and 7 and 14 after the *Mtb* challenge, mice were given CD25 antibody or control rat Ig i.p. Representative data from one of two experiments with 5–7 mice/group are shown. ***p < 0.001, **p < 0.01, *p < 0.05 compared to unimmunized animals with clean water, one-way ANOVA with Tukey's post test.

CD25 depletion alone reduced *Mtb* CFU by 0.6 log_10_, in the lung and *M. abscessus* alone was also protective (Fig. 4A), while inhibiting the protective effect of Ad85A i.d. (Fig. 4B). CD25 depletion had no effect on Ad85A i.d. induced protection but reversed the inhibitory effect of *M. abscessus* on *Mtb* load in the lungs and spleen. Control rat Ig had no effect (Fig. 4B).

These data indicated that depletion of CD4+CD25+Foxp3+ cells reversed the inhibitory effect of *M. abscessus* on Ad85A i.d. induced protection, suggesting that T regs may be involved.

## 4 Discussion

In BALB/c mice Ad85A induces a predominantly CD8 T cell response. Cell transfer experiments and *in vivo* depletion of CD4 T cells suggest strongly that the CD8 T cells are protective against *Mtb* [5,6]. We have used this model to explore the contributions of local and systemic immunity to protection against pulmonary challenge and have shown that local immunity is highly protective, most likely because lung resident T cells can act immediately against *Mtb* at the portal of entry [7]. It is also clear that the localization of immune cells in the lungs after i.n. and i.d. immunization differs and that this may also contribute to the efficacy of i.n. immunization (Figs. 1 and 2) [5,6].

However, because most current clinical trials of subunit *Mtb* vaccines have used parenteral routes of immunization [12] and we observed striking differences in the lung immune response to Ad85A i.d. but not Ad85A i.n. in different environments, here we have focused on the effect of environmental factors on protection induced by parenteral Ad85A immunization. In OTCs in the animal facility we first used, A85A i.d. induced very few lung antigen-specific cells. In contrast in all other OTC and IVC facilities, Ad85A i.d. generated large 85A-specific lung CD8 T cell populations (Fig. 1). Significant protection against *Mtb* was induced in IVCs but was always less than protection conferred by Ad85A i.n. Inhibition of *Mtb* growth occurred late after challenge, as with other parenteral vaccines [8]. Protection was long lasting, in contrast to the reported effect of intra-muscular Ad85A, which conferred protection at 4 but not 12 weeks [20]. Thus the change from OTCs in the original animal house to new conditions correlated with the presence of an increased number of antigen specific T cells in the lungs and improved protection against *Mtb* after Ad85A i.d. immunization.

While immunization with Ad85A i.d. was greatly affected by the environment, immunization with Ad85A i.n was not. Ad85A i.n induces a lung resident self-maintaining protective population and [6,11,21] which includes a BAL population whose presence correlates with inhibition of *Mtb* growth early after challenge [6]. Many BAL cells express CXCR6+ and also bind MHC-85A peptide tetramers [6] but we have not yet determined whether iNKT cells expressing CXCR6 also contribute to early inhibition of *Mtb* growth [22]. These data indicate that the environmental effects of OTCs or IVCs, have a more profound influence on lung homing cells induced by parenteral immunization than the lung resident population induced by i.n. immunization.

We reasoned that the difference in immune responses (Fig. 1A), might be due to varying microbial exposure in different animal houses and might be mimicked by adding mycobacteria to the drinking water concurrently with immunization. This does not prevent entry of 85A-specific T cells into the lungs after i.d. immunization, although the percentage is lower in 2 out of 4 experiments. However, the protective effect of Ad85A i.d. is reduced when BCG or *M. abscessus* are administered orally (Fig. 3). Numbers of CD4+CD25+FoxP3+ T regulatory cells in the lungs, spleen or mesenteric nodes are unaltered after concurrent immunization with Ad85A and oral mycobacteria, although *in vivo* CD25 treatment abolishes the inhibitory effect of oral mycobacteria on Ad85A i.d. induced protection. Similarly unchanged numbers of T regs are observed in other models where they have functional effects [16], so that it remains possible that oral BCG or *M. abscessus* induce T regs that affect lung immune cells. It is also known that T regs can decrease protection against *Mtb* [16].

It is clear that events in the intestine can influence lung immunity. For example, *Helicobacter pylori* infection can prevent the induction of lung allergic disease [9] and the gut microbiota influences the response to lung influenza virus infection [10]. Alterations in both lung dendritic cells and T reg function have been described in these models. In germ free mice increased numbers of CXCR6+ iNKT cells are found in colon and lung due to increased CXCL16 expression and both colitis and lung allergic responses are increased [22]. In other tissues however, fewer iNKT cells are seen in germ free animals. Such effects may also be unique to one animal house. Thus it was found that C57BL/6J mice lacked intestinal Cytophaga-Flavobacteria-Bacteroides (CFB) commensals that induce lamina propria (LP) Th17 cells during development, while all other C57BL/6 mice tested have CFB and a greater number of LP TH17 cells [14]. While we have not demonstrated changes in the microbiota, we have shown that short term exposure to mycobacteria has an effect on lung immunity and that this is organism specific, as illustrated by the different effects of BCG and *M. abscessus* compared to *M. smegmatis*. In addition to microbial effects on adaptive immune responses, parenteral BCG administration alters gene expression in lung stromal components [23], so that several mechanism may contribute to the down-regulation of protective immune responses to Ad85A i.d. observed here.

The variation in BCG effectiveness in different locations [1] has been ascribed to environmental effects, including exposure to EM. Experimentally, parenteral pre-immunization of mice with EM can block induction of protective immunity to *Mtb* by BCG, but did not affect immunity induced by a subunit vaccine [2]. In contrast concurrent oral exposure to mycobacteria, a route more akin to likely exposure in the field, profoundly affected immunity to a parenteral adeno-based subunit vaccine.

We show that an altered environment changes the distribution of immune cells in mice immunized parenterally with this adeno-based subunit vaccine and that the altered distribution correlates with protection after *Mtb* challenge. Furthermore, exposure to oral EM can abolish protective immunity induced by the same subunit vaccine. These results have important implications for the development and application of parenteral subunit vaccines as boosters for use after BCG priming. Better understanding of the mechanisms underlying environmental effects may lead to the development of new strategies to improve the efficacy of such vaccines.
